# Targeting Senescence in Adipose Microvasculature: A New Target Diabetes and Heart Failure

**DOI:** 10.1016/j.jacbts.2026.101557

**Published:** 2026-04-24

**Authors:** Mallikarjun Patil, Daniel J. Tyrrell

**Affiliations:** Department of Pathology, Heersink School of Medicine, University of Alabama at Birmingham, Birmingham, Alabama, USA

**Keywords:** adipose, diabetes, microvasculature, senescence

**Figure:**
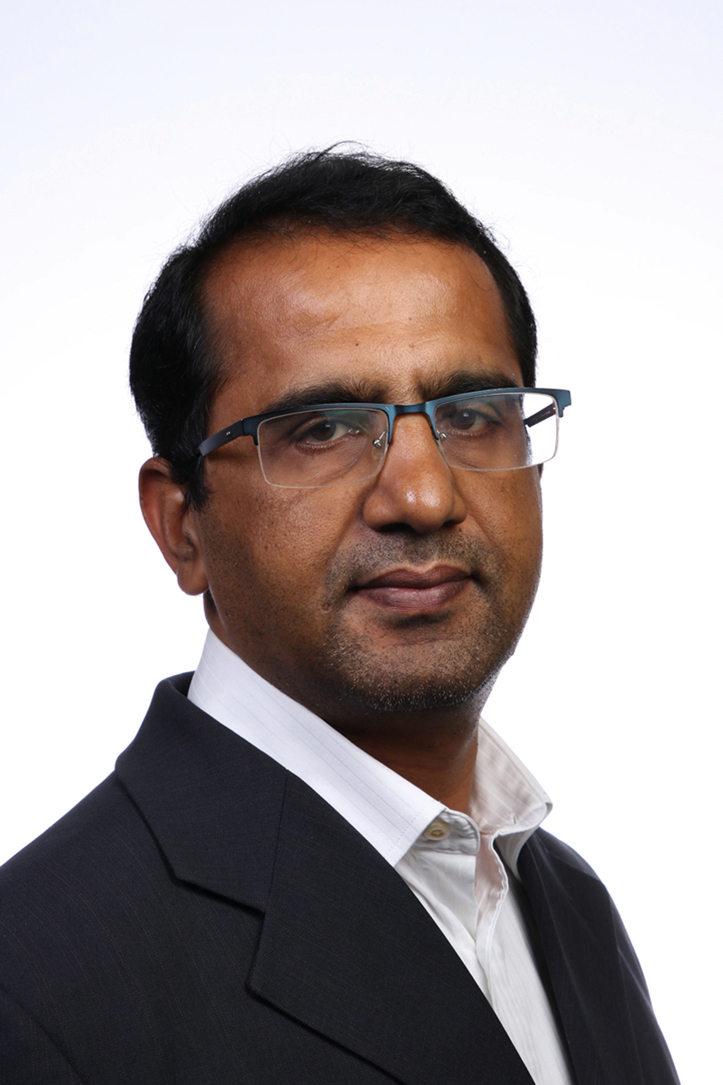

The incidence of diabetes has reached pandemic levels across the globe. More than 40 million Americans have diabetes (90% to 95% have type 2 diabetes mellitus [DM]), and roughly 1 in 3 Americans have pre-diabetes. According to the Centers for Disease Control and Prevention, pre-diabetes is indicated by either a hemoglobin A_1C_ level of 5.7%-6.4% (39-47 mmol/mol, fasting plasma glucose of 100-125 mg/dL [5.6-6.9 mmol/L], or 2-hour oral glucose tolerance test of 140-199 mg/dL [7.8-11.0 mmol/L]). Diabetes is a threat to the lifespan of humans. On average, DM reduces lifespan by 6-14 years depending on the age at diagnosis.^1^ DM predisposes patients to various comorbidities including heart failure (HF). DM increases the risk of HF by 2- to 4-fold, and DM and HF create a positive feedback loop, where one drives the other.^2^

DM also causes microvascular dysfunction (MVD) where tissue perfusion is compromised. Chronic MVD promotes complications of DM, including diabetic nephropathy, retinopathy, and skeletal muscle insulin resistance.^3^ Recent studies have shown that adipose tissue perfusion is also compromised in individuals with DM and those at risk of developing DM.^4^^,^^5^ Tissue perfusion deficits have been identified earlier than onset of hyperglycemia in individuals that develop DM. Adipose tissue MVD in patients with DM has previously been described; however, the molecular mechanisms linking MVD causally to the pathophysiology of DM or HF have remained elusive.

A potential mechanism linking MVD with DM and HF is senescence, which is the permanent exit of a cell from the cell cycle. Cellular senescence is required for maintenance of tissue homeostasis during development and prevention of cells undergoing uninterrupted cell growth and becoming cancerous. Although senescent cells do not divide, they remain metabolically active and release reactive mediators such as reactive oxygen species and proinflammatory cytokines and chemokines.^6^ Cellular senescence is a feature of aging, and the proportion of senescent cells increases with age and has been mechanistically linked to many disease processes.^6^ Individuals with HF and DM tend to be older, multimorbid, frail, and burdened by polypharmacy. Several of the key manifestations of DM, including hyperglycemia, neurohormonal dysfunction, and metabolic shifts, have been identified to promote the increase in accumulation of senescent cells.

The multimorbid nature of many people with DM and HF makes it difficult to implement evidence-based therapies and results in poor quality-of-life outcomes. Current available therapies focus on cardiac and glycemic parameters; however, there are many other potential pathophysiologic mechanisms that could be tractable for emerging therapies. Cellular senescence has been previously described to increase during the pathogenesis of DM and has emerged as a promising potential therapeutic target for DM. In this issue of *JACC: Basic to Translational Science*, the work from Brown et al^7^ begins to tackle some of the current challenges by identifying novel potential future therapeutic targets for HF + DM patients.

Brown et al^7^ identify subcutaneous adipose tissue as a key site regulating metabolic homeostasis, which is critical for glycemic control in HF + DM pathogenesis. They acquired samples from HF and HF + DM patients undergoing surgical placement of cardiac implantable electronic devices and performed a battery of assays to characterize the subcutaneous adipose tissue and microvasculature within.

Brown et al^7^ have identified subcutaneous adipose tissue dysfunction in DM + HF as evidenced by larger-size adipocytes, low number of blood vessels, and defective angiogenesis. Subcutaneous adipose tissue microvessel endothelial cells (SATMVECs) that make up the fat-infiltrating microvasculature from HF + DM patients demonstrated a striking signature of senescence. Endothelial cells from HF + DM patients were characterized by large, swollen cell bodies, increased senescence-associated β-galactosidase staining, low metabolic function, and upregulated senescence-associated secretory phenotype factors including proinflammatory cytokines, interleukin (IL)-6 and IL-8 compared with HF control patients. They also showed that endothelial cells from DM + HF patients have defective tube formation that indicates defective angiogenesis.

Through co-culture experiments combining SATMVECs from patients with either HF or HF + DM and adipocytes derived from healthy donor, Brown et al^7^ found that the endothelial cells from HF + DM patients promoted adipocyte dysfunction. They observed that senescent SATMVECs increased expression of IL-6 and inhibited adiponectin expression. Although the cell surface localization of glucose transporter type 4 was not different between groups, it negatively correlated with hemoglobin A_1C_ and age. Moreover, 2-NBDG (2-[N-(7-nitrobenz-2-oxa-1, 3-diazol-4-yl)amino]-2-deoxy-d-glucose) glucose uptake by the adipocytes co-cultured with endothelial cells from HF + DM patients was significantly inhibited, suggestive of insulin resistance.

Brown et al^7^ further questioned whether endothelial cell senescence can be targeted to improve insulin sensitivity in adipocytes. The authors extended previous findings by demonstrating that digoxin, a cardiac glycoside used clinically for HF, has senolytic properties but only at concentrations greater than the safe therapeutic window for humans.^8^ Brown et al^7^ investigated digoxin has senolytic effects on SATMVEC from DM + HF patients when treated within the therapeutic range. Although modest, the authors identified that digoxin partially reduces senescence in subcutaneous adipose tissue endothelial cells in the HF + DM samples but not in HF samples. They observed that digoxin inhibited IL-6 and IL-8 gene expression, improved cell proliferation, inhibited senescence-associated β-galactosidase activity, and improved metabolism in SATMVECs from DM + HF patients. Furthermore, in co-culture experiments using endothelial cells of HF + DM patients treated with a low dose of digoxin, they showed that 2-NBDG (2-[N-(7-nitrobenz-2-oxa-1, 3-diazol-4-yl)amino]-2-deoxy-d-glucose)-glucose uptake by adipocytes was significantly improved. Despite the modest effect of digoxin, the studies are commendable in their use of a clinically relevant dose.

These studies show the potential for out-of-the-box thinking for therapeutics to treat the highly complex cases associated with HF + DM patients. The ongoing DECISION (Digoxin Evaluation in Chronic heart failure: Investigational Study In Outpatients in the Netherlands) trial using digoxin for HF treatment in diabetes patients should provide a definitive answer for whether digoxin therapy improves outcomes in DM + HF patients.

However, there are several limitations to the study by Brown et al.^7^ The authors demonstrated senescence in vitro and analyzed the expression of retinoblastoma (Rb) at the mRNA level. Although they observed increased expression of total *Rb* mRNA, they did not examine *Rb* phosphorylation. They also did not observe differences in expression in p21, p16, or p53. These genes are critical regulators of the cell cycle and senescence. These would have helped to confirm the senescence-related findings.

Interestingly, SATMVECs from DM + HF patients on metformin were no different from patients not taking metformin. Metformin has been shown to exhibit senostatic and anti-aging effects.^9^ Brown et al^7^ demonstrate improved glucose uptake in their studies but did not investigate insulin-mediated glucose uptake which could have added additional information for cells isolated from individuals who are given metformin. These findings raise several questions: Does metformin have senolytic functions on adipose-derived endothelial cells? Can drugs reach therapeutic levels in the setting of microvascular dysfunction with poor perfusion? Can cellular senescence be reversed in vivo in this clinical population? Digoxin has very narrow therapeutic window and is not well tolerated clinically, which the authors acknowledged would make this specific intervention infeasible clinically.

Using the patient-derived SATMVECs, Brown et al^7^ demonstrate that endothelial senescence drives adipose tissue dysfunction in DM + HF patients. They further show that endothelial cell senescence can be reversed to prevent the adipocyte dysfunction in vitro. This opens new avenues for development of therapies for patients with DM and DM + HF. With several ongoing clinical trials targeting senescence (clinicaltrials.org), these results are very encouraging.

## Funding Support and Author Disclosures

The authors have reported that they have no relationships relevant to the contents of this paper to disclose.
